# Comparison of endoscopic and microscopic techniques for tympanic membrane repair: A longitudinal study

**DOI:** 10.6026/973206300214020

**Published:** 2025-11-15

**Authors:** R. Prem Nivas, Theivanai S, Shivakumar S, Karthikeyan B

**Affiliations:** 1 Department of ENT, Head & Neck Surgery, Vinayaka Mission's Kirupananda Variyar Medical College and Hospitals, Vinayaka Mission's Research Foundation (DU), Chinnasiragapadi, Salem, Tamil Nadu 636308, India

**Keywords:** Myringoplasty, chronic middle ear disease, conductive hearing loss, patient outcomes

## Abstract

Chronic tympanic membrane perforations remain a frequent cause of hearing loss, and the optimal surgical approach for repair continues to be debated. This longitudinal comparative study evaluates graft success, hearing improvement, operative time, and complications between microscopic and endoscopic myringoplasty. Among patients studied, the endoscopic technique yielded greater hearing gain, reduced postoperative morbidity, and shorter hospital stay compared to the microscopic method. Although graft success was slightly higher in the endoscopic group (93.3% vs. 86.7%), complications were markedly fewer. Overall, endoscopic myringoplasty demonstrated superior efficiency, patient comfort, and satisfaction, supporting its advantage over the traditional microscopic approach.

## Background:

Myringoplasty, a surgical procedure to close perforations of the tympanic membrane (TM) for nearly seven decades, has conventionally used an operating microscope and involved postauricular, trans canal and endaural approaches [1]. Even though microscopic myringoplasty through a postauricular approach offers a better view of the field of operation, especially in anterior and large TM perforations or a narrow ear canal, it has disadvantages such as incision scars, aesthetic issues and severe patient discomfort and often requires hair shaving and general anaesthesia [2]. Additionally, the direct-line view of a microscope limits the view and examination of the hidden areas in the middle ear cavity. This has slowly been replaced by endoscopic ear surgery with better visualization without requiring any incisions, which reduces the invasiveness and postoperative pain associated with microscopic techniques. This transition started with the introduction of middle ear endoscopy in 1967, followed by advances in the 1990s, which included a wider view with high-resolution endoscopic systems [3].

In contrast to microscopes, endoscopes provide surgeons the ability to visualize structures within the tympanic cavity, including the anterior epitympanum, attic region, sinus tympani, facial recess and hypo tympanum, areas that are inaccessible through the use of a microscope [4]. For educational purposes, endoscopic surgery is also extremely useful in deepening the knowledge of surgical anatomy and procedures by both surgeons and residents [5]. Additionally, in the treatment of cholesteatomas, use of an endoscope reduces the potential for residual disease and recurrence. Since endoscopic ear surgery is less invasive and thus potentially shorter, the operating times, it still presents challenges because it is single-handed; a challenge that potentially could be made in managing significant bleeding and that has a risk of damaging the middle ear components due to the thermal effect of the light source [6]. With this background, the objective of the current study was to compare graft success, hearing outcomes and operating times for patients who received endoscopic versus microscopic myringoplasty [7]. Thereafter, we examined whether the endoscopic approach was superior to the conventional microscopic surgery.

## Materials and Methods:

This was a prospective, longitudinal follow-up study conducted in the Department of Ear, Nose and Throat (ENT), Vinayaka Mission's Kirupananda Variyar Medical College and Hospital, a tertiary health facility, Salem, Tamil Nadu, India, over a period of one year between June 2020 and May 2021. It had prior approval by the Institute Human Ethics Committee. Content of Participant Information Sheet (PIS) in local language was provided to the participants (and their attenders) and contents were read to them in their own language to their satisfaction. The participants were enrolled in the study after obtaining written informed consent. All patients of age 18-60 years presenting to the outpatient department with ear discharge and diagnosed as chronic middle ear disease with conductive hearing loss are included in this study. Patients with comorbidities such as diabetes mellitus, immunocompromised status, patients requiring revision surgery and those with complications of chronic otitis media are excluded from the study. The study population was selective and we had to adopt purposive sampling - convenient sampling technique; all patients reporting to the outpatient department were taken for the study period were randomly allotted (simple randomization technique) to two groups; 30 patients in each group; Group A patients underwent conventional microscope assisted middle ear surgery and Group B patients underwent endoscope assisted middle ear surgery.

Patients were followed up for six months at regular intervals. Patients were not aware of the group or type of intervention single blinding was done. A purpose predesigned, semi structured and pretested questionnaire was used to collect sociodemographic characteristics, detailed history and record general physical examination, clinical examination findings of the ear and mastoids. Pure-tone average audiometry was done before and after surgery (6 months) with a clinical audiometer. Anatomic success was defined as complete closure of TM perforation without medialization, lateralization, or perforation. An ABG of less than or equal to 10 dB was accepted as functional success. An X-ray mastoid (Law's view) was taken for all patients and CT-mastoids for patients when warranted. Surgical technique: Myringoplasties were performed on all patients who underwent surgery under general anesthesia; in the microscopic group, they used Möller-Wedel Optical equipment produced in Hamburg, Germany, whereas in the endoscopic group, they used equipment from Karl Storz, of Tuttlingen, Germany. In the microscopic procedure, it was done in a post-auricular approach and they de-epithelized the edges in a circular form. Then the canal skin flap was elevated to view the middle ear. If the anterior canal wall was bulging, canaloplasty was performed using a diamond drill. Ossicular movement was observed by noting the round window reflex. Tragal cartilage was harvested and was prepared as a graft. The perichondrium was intact on both sides during the process of preparation. The 0-degree rigid endoscope and the endoscopic system were used in the endoscopy group. As in the microscopic procedure, in the endoscopy technique, an incision was made that de-epithelized circularly around the edges of perforation and a vertical cut 5 mm laterally to the annulus. This incision was then connected with radial incisions at 6 and 12 o'clock. The tympanomeatal skin flap was elevated to visualize the middle ear and the movement of ossicles was evaluated according to the round window reflex. The technique for harvesting and preparing the graft was identical to that in Group 1. The hand-collected data was entered into a Microsoft Excel sheet and then passed through Stata 16 for analysis. For categorical variables, the descriptive analysis used numbers and percentages. The mean and standard deviation or median and interquartile range were applied for continuous variables. Chi-square test of significance two-sided was used to assess for association for categorical variables. In situations where the expected values in the cells were less than five in 20% of the cells, Fisher's exact test two-sided was used. Use independent "t" tests of two-sided direction to test whether the continuous independent variables are significantly associated. Statistical significance is taken at p<0.05.

## Results:

This study assessed the outcome of endoscopic (Group B) versus microscopic (Group A) myringoplasty in patients with chronic middle ear disease and conductive hearing loss. In fact, Group B obtained significantly superior outcomes in hearing compared to the group: there were significantly more hearing gain achieved (12.5 ± 1.3 vs. 11.7 ± 0.7, p=0.004), less postoperative air conduction (25.8 ± 4.9 dB vs. 29.9 ± 7.2 dB, p=0.013), with reduction of the ABG comparable for both groups postoperatively. Operating time was significantly shorter with endoscope-assisted surgery (58.7 ± 11.2 minutes vs. 79.1 ± 16.3 minutes, p<0.001) and shorter hospital stay (2.9 ± 2.1 days vs. 6.2 ± 1.9 days, p<0.001). Group B patients experienced less postoperative pain (VAS 4.6 ± 0.9 vs. 8.8 ± 0.7, p<0.001) and complications (6.7% vs. 26.7%, p=0.038). Graft success (93.3% vs. 86.7%), functional success (76.7% vs. 66.7%) and patient satisfaction rates were all higher in Group B; however, the difference was not statistically significant. Patient satisfaction was significantly higher in Group B (60.2% vs. 43.9% in Group A). The demographic and clinical profiles of patients in both groups were comparable, ensuring a fair basis for outcome comparison (Table 1 - see PDF). Endoscopic myringoplasty has significant advantages over microscopic myringoplasty, such as less postoperative pain, shorter hospital stays, faster operating time, better hearing improvement and fewer complications (Table 2 - see PDF). Table 3 (see PDF) Comparable graft and functional success rates between the groups, though Group B had slightly higher percentages. Overall, these findings underscore the efficiency, safety and enhanced patient outcomes associated with endoscopic myringoplasty. Table 4 (see PDF) Profile of Postoperative Complications in Microscopic vs. Endoscopic Myringoplasty. Postoperative complications included wound-related issues, localized infections, aesthetic discomfort (asymmetry) and soft tissue complications. Data based on 30 patients in each group.

The results tables collectively highlight the superior outcomes of endoscopic (Group B) over microscopic (Group A) myringoplasty. Confirmation of comparable baseline sociodemographic and clinical characteristics between the groups is shown by Table 1 (see PDF), while Table 2 (see PDF) demonstrates significantly better outcomes in Group B, with higher hearing gain, operating times also being shorter, with reduced postoperative air conduction and shorter hospital stays, significantly less postoperative pain and fewer complications. Table 3 (see PDF) Compares graft and functional success rates between the groups, though Group B had slightly higher percentages. Overall, these findings underscore the efficiency, safety and enhanced patient outcomes associated with endoscopic myringoplasty. Table 4 (see PDF) reveals a markedly lower incidence of postoperative complications in the endoscopic group (6.7%) compared to the microscopic group (26.7%). While minor infections and postauricular pain occurred in both groups, wound gap and pinna asymmetry were exclusively seen in Group A. Notably, no cases of ear stenosis were reported in either group. These findings support the less invasive nature of endoscopic myringoplasty, which likely contributed to reduced tissue trauma and enhanced healing outcomes. Figure 1 (see PDF) below is the radar chart representing patient satisfaction of microscopic (Group A) and endoscopic (Group B) myringoplasty. Group B exhibited greater satisfaction levels, with fully satisfied patients of 60.2% whereas in Group A 43.9%. Moreover, the number of partially satisfied (30.7% vs. 40.7%) or not satisfied (9.1% vs. 15.4%) patients in Group B were less than those in Group A. This is evidence of the fact that endoscopic myringoplasty resulted in better overall patient satisfaction than microscopic myringoplasty. Figure 1 (see PDF) represents higher patient satisfaction in Group B, with 60.2% reporting full satisfaction compared to 43.9% in Group A and fewer patients in Group B expressing dissatisfaction.

## Discussion:

The character of ear surgery changed after the introduction of operating microscopes. Today, many otological procedures are performed with both hands under an operating microscope, which provides the surgeon with stereoscopic vision and bimanual handling [16]. A study of 60 patients with chronic middle ear disease and conductive hearing loss, showed that distinct advantages were realized with endoscope-assisted middle ear surgery, Group B compared to microscope-assisted, Group A [8]. Demographic analysis showed similar tympanic membrane perforations, but in Group B higher hearing gains (12.5 vs. 11.7 dB, p<0.05) and lower postoperative air conduction (25.8 vs. 29.9 dB, p=0.013) [9]. Group B patients also had lower postoperative pain, shorter hospital stays and faster recovery, supported by a shorter mean operating time (58.7 vs. 79.1 minutes, p<0.001) [10]. Graft and functional success rates were comparable, but complications were significantly fewer in Group B (6.7% vs. 26.7%, p<0.05), emphasizing the safety of endoscopic techniques. Enhanced visualization and minimally invasive approaches contributed to better precision and reduced tissue trauma [11, 12]. Patient satisfaction was much higher in Group B, where 60.2% of patients were fully satisfied, as compared to 43.9% in Group A, indicating the overall benefits of endoscopic methods [13, 14]. The above results point out that endoscope-assisted surgery is superior to other approaches regarding auditory outcomes, recovery and patient experience [15].

## Conclusion:

Endoscope-assisted middle ear surgery offered significant benefits over conventional microscope-assisted surgery in terms of improved hearing outcome, shorter time taken for operation, fewer complications and patient satisfaction. In comparison, the two approaches shared equal graft success and functional results, thus ensuring both are useful for chronic middle ear disease treatment. Thus, endoscope-assisted surgery could improve patients' outcomes, hence optimizing resource utilization in the health sector. In addition, there is the emphasis of individualization of surgical choice.
